# Comparison of Fusion Rates among Various Demineralized Bone Matrices in Posterior Lumbar Interbody Fusion

**DOI:** 10.3390/medicina60020265

**Published:** 2024-02-02

**Authors:** Sanghoon Lee, Dae-Woong Ham, Ohsang Kwon, Joon-Hee Park, Youngsang Yoon, Ho-Joong Kim

**Affiliations:** 1 Department of Orthopedic Surgery, Seoul National University Bundang Hospital, Seoul National University College of Medicine, Seongnam-si 13620, Republic of Korea; sanghoon92@gmail.com (S.L.); ormssang@gmail.com (O.K.); 2 Department of Orthopaedic Surgery, Chung-Ang University Hospital, Chung-Ang University College of Medicine, Seoul 06974, Republic of Korea; hamdgogo@cau.ac.kr; 3 Department of Anesthesiology and Pain Medicine, Kangdong Sacred Heart Hospital, Hallym University College of Medicine, Seoul 05355, Republic of Korea; junhee93@kdh.or.kr (J.-H.P.); 200341@kdh.or.kr (Y.Y.)

**Keywords:** demineralized bone matrix, posterior lumbar interbody fusion, fusion rate, Bridwell grade

## Abstract

*Background and Objectives*: Posterior lumbar interbody fusion (PLIF) plays a crucial role in addressing various spinal disorders. The success of PLIF is contingent upon achieving bone fusion, as failure can lead to adverse clinical outcomes. Demineralized bone matrix (DBM) has emerged as a promising solution for promoting fusion due to its unique combination of osteoinductive and osteoconductive properties. This study aims to compare the effectiveness of three distinct DBMs (Exfuse^®^, Bongener^®^, and Bonfuse^®^) in achieving fusion rates in PLIF surgery. *Materials and Methods*: A retrospective review was conducted on 236 consecutive patients undergoing PLIF between September 2016 and February 2019. Patients over 50 years old with degenerative lumbar disease, receiving DBM, and following up for more than 12 months after surgery were included. Fusion was evaluated using the Bridwell grading system. Bridwell grades 1 and 2 were defined as ‘fusion’, while grades 3 and 4 were considered ‘non-fusion.’ Clinical outcomes were assessed using visual analog scale (VAS) scores for pain, the Oswestry disability index (ODI), and the European quality of life-5 (EQ-5D). *Results*: Fusion rates were 88.3% for Exfuse, 94.3% for Bongener, and 87.7% for Bonfuse, with no significant differences. All groups exhibited significant improvement in clinical outcomes at 12 months after surgery, but no significant differences were observed among the three groups. *Conclusions*: There were no significant differences in fusion rates and clinical outcomes among Exfuse, Bongener, and Bonfuse in PLIF surgery.

## 1. Introduction

Lumbar degenerative disease is a prevalent condition that often requires spinal surgery, representing the general term for various ailments characterized by clinical symptoms like lower back pain and leg pain. These symptoms arise from the degenerative alterations in the articular cartilage and ligaments of the lumbar intervertebral disc and facet joint. Surgical intervention aims to achieve decompression, reduction, fusion, and stabilization. Lumbar interbody fusion (LIF) emerges as a crucial method for comprehensive decompression and the restoration of spinal function, significantly enhancing spinal stability and elevating the safety and efficacy of surgical procedures. Among the well-established techniques for treating lumbar degenerative diseases, posterior lumbar interbody fusion (PLIF) stands out as a mature approach with confirmed therapeutic benefits [1,2,3]. Failure to achieve bone fusion in this surgery leads to several complications, including pseudarthrosis, implant subsidence, and pedicle screw loosening [4,5]. Additionally, this instability can result in worse clinical outcomes. Therefore, the ultimate aim of PLIF surgery is to achieve solid fusion.

To achieve solid fusion in PLIF surgery, it is necessary to use effective bone graft materials. The preferred choice involves using an autologous iliac bone graft due to the substantial volume of cancellous bone available from the inner table of the pelvis, which provides all the necessary graft characteristics. Despite being considered the gold standard, the use of autologous iliac bone grafts is associated with significant morbidity rates. Studies have reported high rates, up to 40%, of persistent donor site complications such as pain, paresthesia, hematoma, and infection [6]. Exploring alternatives to autologous iliac bone graft, including local autograft, calcium-phosphate salts, demineralized bone matrix (DBM), the bone morphogenetic proteins (BMP) family, autogenous growth factors, bone marrow aspirate, and collagen-based matrices, is becoming increasingly popular. These alternatives are gaining acceptance in PLIF procedures. Among them, DBM has emerged as a promising contributor to this field, owing to its unique combination of osteoinductive and osteoconductive properties [7,8]. DBM is typically obtained from allograft bone, which is harvested from human donors. The bone undergoes a process of demineralization, during which mineral components, such as calcium, are removed. This results in a matrix that retains the organic and proteinaceous components of bone, including growth factors, collagen, and other proteins. This matrix offers a biological substrate that aids in new bone formation and integration. Due to its allogenic nature, DBM reduces the risk of complications associated with autologous iliac bone grafts. Numerous animal studies have reported promising outcomes. Some rat studies, for instance, have indicated that DBM can induce spinal fusion in a dose-dependent manner, suggesting its potential as a graft substitute or extender. In a canine study, the combination of DBM with autograft demonstrated faster spinal fusion compared to autograft alone, and in a rabbit interbody fusion model, a composite graft of DBM and hydroxyapatite block exhibited quicker and stronger fusion than autograft alone [9,10]. Furthermore, numerous human studies have demonstrated that the utilization of DBMs is an effective choice for bone union in PLIF surgery [11,12].

Several DBM products are currently available and extensively utilized in PLIF surgery. Despite their widespread use, there is a notable absence of prior studies that have compared their effectiveness. Therefore, we conducted a comparison of fusion rates among three distinct DBMs using computed tomography (CT) scans at the 12-month mark following PLIF surgery. Additionally, we examined clinical outcomes among the three DBMs after surgery.

## 2. Materials and Methods

### 2.1. Study Design and Patients

This retrospective review was conducted on patients undergoing PLIF at a single institution and was approved by the institutional review board of Seoul National University Bundang Hospital (B-2004-604-107). A total of 236 consecutive patients who underwent PLIF between September 2016 and February 2019 were included in this study. The inclusion criteria were as follows: (1) age >50 years; (2) diagnosed with degenerative lumbar disease with subsequent PLIF using DBM; (3) follow-up and CT scan performed more than 1 year after surgery. The exclusion criteria were as follows: (1) patients with osteoporosis, indicated by a lumbar spine L1-4 mean T-score < −4.0 on a dual-energy X-ray absorptiometry (DEXA) bone density scan; (2) patients with fractures, infectious diseases, or malignant tumors in the lumbar region to be operated on; (3) patients with abnormal blood calcium and phosphorus levels exceeding 30% of the upper and lower limits of the normal range; (4) patients with an unclear medical history. The diagnosis was based on clinical symptoms, plain radiographs, CT, and magnetic resonance imaging (MRI).

The PLIF in this study was conducted by one experienced spine surgeon (H-J.K, with 20 years of experience). With general anesthesia, a midline incision was made along the lower back, exposing the target vertebrae. The muscles and soft tissue were gently removed to provide access to the spine. Initially, rigid pedicle screw fixation was performed. To achieve thorough decompression, procedures including laminectomy, facet joint resection, disc removal, and end plate preparation were performed. The soft tissue surrounding the local bone and the cartilage portion were removed, and the entire local bone fragment was combined with 5 mL of DBM. This bone graft material was then inserted into the disc space. A polyetheretherketone cage filled with bone graft material was placed between the vertebrae to restore disc height and facilitate fusion. Rods were inserted to provide stability. The DBMs used for comparison in this study were (1) ExFuse^®^ (Hans Biomed, Seoul, Republic of Korea), (2) Bongener^®^ (CG Bio, Seoul, Republic of Korea), (3) Bonfuse^®^ (CG Bio, Seoul, Republic of Korea). All three DBMs are putty-type. According to the type of DBM, patients were categorized into three groups: the Exfuse group, the Bongener group, and the Bonfuse group.

### 2.2. Outcome Measurements

The patients were routinely followed up with plain radiographs that included flexion/extension lateral radiographs at 3, 6, and 12 months postoperatively. Additionally, a 1 mm slice coronal and sagittal CT was assessed to more accurately evaluate the fusion status at 6 and 12 months postoperatively. Interbody fusion rates were assessed with the Bridwell grading system (grade 1, fused with remodeling and trabeculae present; grade 2, not fully remodeled and incorporated, but no lucency present; grade 3, potential lucency present at the top and bottom of the graft; grade 4, fusion absent with collapse/resorption of the graft). In this study, Bridwell grades 1 and 2 were defined as ‘fusion’, while grades 3 and 4 were considered ‘non-fusion.’ The grading was determined based on measurements by two experienced spin surgeons in a blinded manner (D.H and S.L).

To assess clinical outcome, lower back pain and leg pain were evaluated using a visual analog scale (VAS, on a scale of 0 to 10, with higher scores indicating more severe pain). In addition, self-reported questionnaires, including the Oswestry disability index (ODI) and the European quality of life-5 (EQ-5D), were collected. The ODI is a widely used questionnaire designed to assess the level of disability or functional impairment experienced by individuals with low back pain. The ODI comprises 10 questions assessing patients ability to handle daily activities, encompassing pain intensity, lifting, self-care, walking, sitting, sexual function, standing, social life, sleep quality, and travel ability. Each question has six statements, scored from 0 to 5, with patients selecting the statement that best aligns with their ability. The index is calculated by summing all question scores and multiplying by two. The resulting score/index ranges from 0 to 100, indicating different levels of disability: 0–20 reflects minimal disability, 21–40 moderate disability, 41–60 severe disability, 61–80 crippled, and 81–100 bed-bound. The EQ-5D score is a standardized instrument used to measure health-related quality of life. It assesses an individual’s general health and well-being and is commonly used in healthcare and clinical research to evaluate the overall health status and quality of life of patients. The descriptive system includes five dimensions: mobility, self-care, usual activities, pain/discomfort and anxiety/depression. Each dimension presents five levels: no problems, slight problems, moderate problems, severe problems, and extreme problems. Patients are required to mark the box adjacent to the most suitable statement for each of the five dimensions to indicate their health state. This selection yields a 1-digit number representing the chosen level for that dimension. The digits from all five dimensions can be assembled into a 5-digit number, offering a description of the patient’s overall health state.

### 2.3. Statistical Analysis

The baseline characteristics and clinical outcomes for continuous variables were compared among the three groups using the Student’s *t* test. The chi-square test and Fisher’s exact test are used for analyses of categorical variables. *p*-values < 0.05 were considered statistically significant. Statistical analyses were performed using SPSS (version 27.0; IBM Corp., Armonk, NY, USA).

## 3. Results

A total of 236 patients in the three groups, with operations on 290 segments, were followed up for 12 months. The Exfuse group consisted of a total of 98 patients, with operations on 103 segments. The Bongener group consisted of 72 patients, with operations on 106 segments. The Bonfuse group consisted of 66 patients, with operations on 81 segments. The mean age at the time of surgery was 67.3, 69.9, and 69.4 in the Exfuse, Bongener, and Bonfuse groups, respectively. The majority of cases involved a one-level fusion, with most of the single-level fusions performed at the L4–5 level in all three groups. The mean number of levels was 1.05 in the Exfuse group, 1.47 in the Bongener group, and 1.23 in the Bonfuse group. The groups had similar average ages at the time of surgery (67.3 ± 7.1 vs. 69.9 ± 8.5 vs. 69.4 ± 7.7 years, respectively, *p* = 0.077) and sex distribution (35.7% vs. 34.7% vs. 24.2% proportion of male, respectively, *p* = 0.262). Demographic and baseline characteristics were not significantly different among the three groups except for height. In addition, all clinical outcome values at the preoperative stage were not significantly different among the three groups (Table 1).

**Figure 1:**
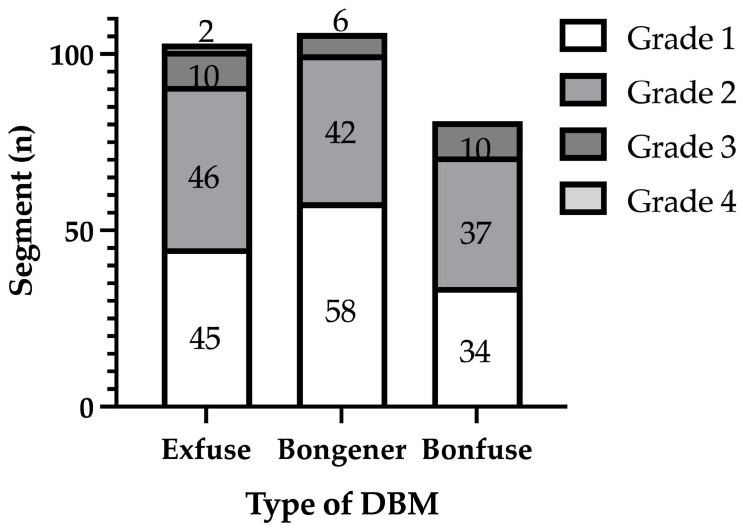
The Bridwell grade of interbody fusion among the three groups.

**Figure 2:**
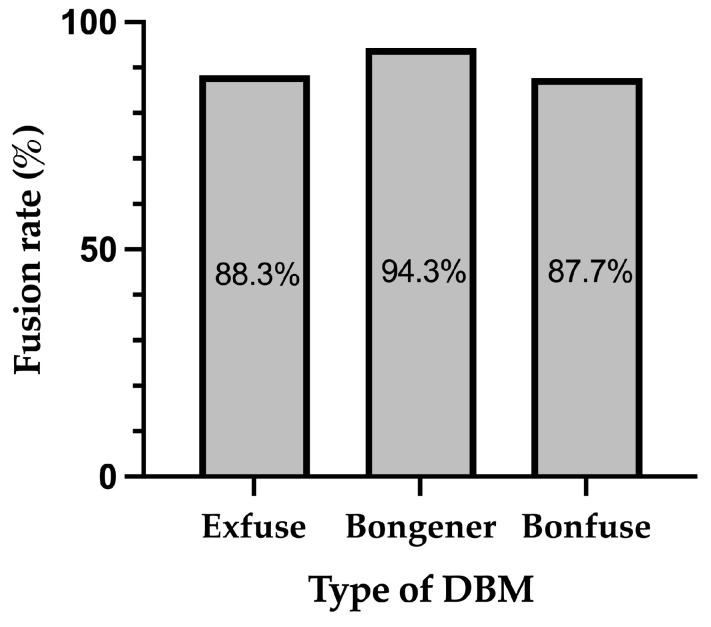
Comparison of Fusion Rate among the three groups.

Figure 1 shows the distribution of segments across different Bridwell grades at 12 months after surgery within three distinct groups: Exfuse (*n* = 103), Bongener (*n* = 106), and Bonfuse (*n* = 81). In the Exfuse group, there were 45 segments with grade 1, 46 segments with grade 2, 10 segments with grade 3, and 2 segments with grade 4. In the Bongener group, there were 58 segments with grade 1, 42 segments with grade 2, and 6 segments with grade 3. In the Bonfuse group, there were 34 segments with grade 1, 37 segments with grade 2, and 10 segments with grade 3. The difference among the three groups in the distribution of Bridwell grade at 12 months after surgery was not statistically significant (*p* = 0.203). Figure 2 displays the fusion rates among the three groups. The Exfuse group exhibited a fusion rate of 88.3%, the Bongener group 94.3%, and the Bonfuse group 87.7%, with no significant differences in the fusion rate among the three groups. (*p* = 0.214).

The preoperative VAS scores for back pain were 6.05 ± 2.46, 6.41 ± 2.55, and 6.40 ± 2.50 in the Exfuse, Bongener, and Bonfuse groups, respectively. At 12 months after surgery, the VAS scores were 4.70 ± 2.89, 4.45 ± 2.40, and 5.07 ± 3.33 for the three groups. Similarly, the preoperative VAS scores for leg pain were 6.74 ± 2.34, 6.74 ± 2.60, and 7.02 ± 2.69, and at 12 months after surgery, they were 3.49 ± 3.19, 4.49 ± 2.21, and 4.81 ± 3.64, respectively. In all three groups, VAS scores for back and leg pain at 12 months after surgery showed statistically significant improvements compared with baseline (*p* < 0.01 for both). The preoperative ODI scores were 20.93 ± 7.88, 22.67 ± 8.64, and 22.83 ± 7.36 for the Exfuse, Bongener, and Bonfuse groups, respectively. At 12 months, the ODI scores improved to 14.29 ± 9.26, 16.42 ± 7.60, and 17.07 ± 10.34, respectively. For the preoperative EQ-5D scores, the values were 0.338 ± 0.266, 0.303 ± 0.300, and 0.348 ± 0.279 for the Exfuse, Bongener, and Bonfuse groups, respectively. After 12 months, the EQ-5D scores changed to 0.504 ± 0.328, 0.494 ± 0.264, and 0.416 ± 0.350, respectively. Both ODI and EQ-5D scores improved significantly in each group compared with baseline (*p* < 0.01 for both). However, there were no significant differences in the changes observed in VAS scores for back and leg pain from baseline to 12 months after surgery, as well as the ODI and EQ-5D scores, among the three groups (Table 2).

Lastly, the comparison of clinical outcomes between the fusion and non-fusion groups is presented in Table 3. At 12 months after surgery, VAS scores for back pain in the fusion/non-fusion group were 4.64 ± 2.92/5.08 ± 2.75, 4.53 ± 2.41/3.40 ± 2.30, and 4.87 ± 3.30/6.00 ± 3.50 for the Exfuse, Bongener, and Bonfuse groups, respectively. VAS scores for leg pain in the fusion/non-fusion groups were 3.26 ± 3.13/5.00 ± 3.33, 4.35 ± 2.69/6.20 ± 2.68, and 4.85 ± 3.63/4.60 ± 3.86 for the Exfuse, Bongener, and Bonfuse groups, respectively. The ODI scores in the fusion/non-fusion groups were 13.77 ± 8.93/17.58 ± 11.04, 16.34 ± 7.75/17.40 ± 6.07, and 17.40 ± 10.76/15.60 ± 8.55. The EQ-5D scores in the fusion/non-fusion groups were 0.505 ± 0.334/0.500 ± 0.307, 0.502 ± 0.267/0.400 ± 0.234, and 0.408 ± 0.354/0.454 ± 0.344, respectively. No significant differences were observed in all clinical outcome variables across the three DBMs and the entire patient cohort (Table 3).

There were no serious complications in our study population during the follow-up period. Additionally, no patients underwent revision surgery due to nonunion during follow-up.

## 4. Discussion

This study aimed to compare the fusion rates and clinical outcomes among three distinct DBMs, namely Exfuse, Bongener, and Bonfuse, in patients undergoing PLIF surgery. The analysis of the Bridwell grades revealed that the Exfuse group exhibited a fusion rate of 88.3%, the Bongener group 94.3%, and the Bonfuse group 87.7% without significant differences. In terms of clinical outcome variables, all groups exhibited significant improvement in all measures at 12 months after surgery. However, there were no significant differences in the improvements in all measures among the three groups.

The achievement of a fully solid fusion mass is crucial for the positive outcomes and success of lumbar interbody fusion surgery [13,14,15]. Previous literature indicates a radiologic fusion rate for PLIF ranging from 71% to 96% [11,16,17]. Autologous iliac bone graft is considered the gold standard for PLIF surgery [18], but it carries the risk of complications such as donor site morbidity. By avoiding these drawbacks, the use of local bone and DBM is advantageous and has shown good results [14,19]. The mineralized bone matrix itself is not highly osteoinductive, but after the acidic extraction of the bone to eliminate the mineralized components, the bone matrix exhibits a strong ability to differentiate bone progenitor cells into osteoblasts. This phenomenon was initially identified by Urist when he detected bone-forming substances in extracts of demineralized bone, leading to extensive research and development in this field [20]. The fundamental osteoinductive components of DBM comprise small amounts of glycoproteins in the organic component of bone, with the most crucial one named bone morphogenetic protein (BMP) by Urist [21,22]. In addition to its osteoinductivity, DBM exhibits osteoconductivity due to the presence of collagenous and noncollagenous proteins, which remain as osteoconductive material following the demineralization process [23]. Since its introduction in 1991, DBM has found widespread use in spinal fusion surgery. The production of DBM involves processing allogeneic bone from hospitals or tissue banks through various methods, and it is applied with different types of carriers. The properties of DBM significantly depend on the method of its preparation. This encompasses techniques for demineralization (such as solvent, temperature, demineralization duration, and concentration of active components), methods for sterilization (such as gamma irradiation), storage conditions (including temperature), and donor age. Consequently, due to this variability, the overall quantity of BMPs and other crucial growth factors essential for the osteoinductive properties of DBM has been observed to vary widely among different products. These factors contribute to elucidating some of the discrepancies between preclinical and clinical data regarding the osteoinductive potential of DBM [24]. Multiple articles indicate the effectiveness of DBM in lumbar fusion cases, particularly as a local autograft extender. However, concerning interbody fusion, limited studies have directly compared DBM with autografts in the lumbar spine. In a study by Kim et al., HA-DBM (Bonfuse, CG Bio Inc.) was compared with local autograft as an interbody cage filler [25]. No statistically significant differences in functional outcomes were observed at the 12-month mark, and there was no significant distinction in the fusion rate on CT at one year (52% DBM versus 62% local autograft). The authors concluded that HA-DBM demonstrates similar fusion rates to local autograft as an interbody filler. Additionally, Ahn et al. explored the application of DBM as an LA extender in PLIF cages [12]. In a case-control study, 70 patients underwent PLIF with local autograft, while 44 had PLIF with local bone and a DBM extender (Allomatrix). No differences were noted in ODI scores at the 2-year follow-up, and there was no significant distinction in the degree of bone formation between the groups at the 2-year follow-up. In this study, the fusion rates for all three DBMs reported in this study are comparable to those reported in previous studies. Therefore, all three DBMs have the potential to replace autologous iliac bone grafts.

In many previous comparative studies, there were no significantly different clinical outcomes according to the bone graft materials used [26,27,28]. Similarly, in this study, all measurements after surgery showed a significant improvement, but there were no significant differences among the three groups. Moreover, there is a debate regarding the assumption that achieving successful fusion leads to positive clinical outcomes. In certain instances, successful fusion may be associated with pain, whereas patients experiencing non-unions may not necessarily report pain. Fischgrund et al. observed successful arthrodesis in 83% of lumbar fusion surgeries. However, they found that the achievement of successful fusion did not reliably predict positive patient outcomes [29]. Another study by Herkowitz et al. indicated a pseudarthrosis rate of approximately 36%, yet clinical results were excellent [30]. The study concluded that the development of a fibrous union seemed to offer adequate structural support, preventing the progression of spondylolisthesis. Similarly, in this study, no differences in clinical outcomes were observed between the fusion and non-fusion groups. This finding aligns with the study by Park et al., which reported no difference in postoperative back pain, radiating pain, or disability between radiographic solid fusion and non-union groups [31]. The lack of significant differences is likely attributed to the fact that even if a solid fusion is not achieved, the resulting instability is generally not severe enough to cause clinically significant back pain or disability, thereby obviating the need for revision surgery.

There are several limitations to this study. Firstly, given its retrospective design, the data quality was lower compared to a prospective study. Future prospective studies are essential to accurately compare the effectiveness of DBMs. Secondly, the follow-up period was relatively short; we only observed outcomes over 12 months after surgery. However, it is not uncommon for lumbar spinal fusion to require more than a year to achieve complete fusion. Third, the amount of local bone graft used with DBM in each surgery was not measured; however, it is unlikely that there was a significant difference between patients because the amount of local bone available during PLIF surgery did not vary significantly among patients, and most of the local bone obtained was used as bone graft. Finally, the proportions of components in the three DBMs could differ across the manufacturing processes, but we did not conduct an analysis of this because the detailed manufacturing process and components are confidential and difficult to obtain information about.

## 5. Conclusions

This study compared fusion rates and clinical outcomes among three different DBMs—Exfuse, Bongener, and Bonfuse-in patients undergoing PLIF surgery. Fusion rates, assessed by Bridwell grades, were 88.3% for Exfuse, 94.3% for Bongener, and 87.7% for Bonfuse, with no significant differences observed. Furthermore, there were no significant differences in clinical outcome improvements among the three groups.

## Figures and Tables

**Table 1:** Demographic and baseline characteristics.

	Exfuse *n* = 98	Bongener *n* = 72	Bonfuse *n* = 66
Age (years)	67.3 ± 7.1	69.9 ± 8.5	69.4 ± 7.7
Sex (Male/Female)	35/63	25/47	16/50
Height, cm	158.72 ± 8.44	156.43 ± 8.60	155.32 ± 8.29
Weight, kg	63.06 ± 10.17	60.45 ± 11.25	60.38 ± 11.54
BMI, kg/m^2^	24.96 ± 3.09	24.62 ± 3.35	24.94 ± 3.80
Mean number of levels fused (*n*)	1.05	1.47	1.23
VAS ^a^ for back pain	6.05 ± 2.46	6.41 ± 2.55	6.40 ± 2.50
VAS for leg pain	6.74 ± 2.34	6.74 ± 2.60	7.02 ± 2.69
ODI score ^b^	20.9.3 ± 7.88	22.67 ± 8.64	22.83 ± 7.36
EQ-5D ^c^	0.338 ± 0.266	0.303 ± 0.300	0.348 ± 0.279

^a^ Visual analogue scale; ^b^ Oswestry disability index; ^c^ European Quality of life-5.

**Table 2:** Comparison of clinical outcomes among the three groups.

	Exfuse	Bongener	Bonfuse	*p*-Value
VAS ^a^ for back pain				0.307
Baseline	6.05 ± 2.46	6.41 ± 2.55	6.40 ± 2.50	
At 12 months	4.70 ± 2.89	4.45 ± 2.40	5.07 ± 3.33	
VAS for leg pain				0.065
Baseline	6.74 ± 2.34	6.74 ± 2.60	7.02 ± 2.69	
At 12 months	3.49 ± 3.19	4.49 ± 2.21	4.81 ± 3.64	
ODI ^b^				0.891
Baseline	20.93 ± 7.88	22.67 ± 8.64	22.83 ± 7.36	
At 12 months	14.29 ± 9.26	16.42 ± 7.60	17.07 ± 10.34	
EQ-5D ^c^				0.573
Baseline	0.338 ± 0.266	0.303 ± 0.300	0.348 ± 0.279	
At 12 months	0.504 ± 0.328	0.494 ± 0.264	0.416 ± 0.350	

^a^ Visual analogue scale; ^b^ Oswestry disability index; ^c^ European Quality of life-5.

**Table 3:** Comparison of clinical outcomes 12 months after surgery based on fusion status.

	Fusion	Non-Fusion	*p*-Value
VAS ^a^ for back pain			
Exfuse	4.64 ± 2.92	5.08 ± 2.75	0.620
Bongener	4.53 ± 2.41	3.40 ± 2.30	0.314
Bonfuse	4.87 ± 3.30	6.00 ± 3.50	0.336
Total	4.66 ± 3.02	5.11 ± 3.02	0.448
VAS for leg pain			
Exfuse	3.26 ± 3.13	5.00 ± 3.33	0.079
Bongener	4.35 ± 2.69	6.20 ± 2.68	0.145
Bonfuse	4.85 ± 3.63	4.60 ± 3.86	0.845
Total	4.03 ± 3.18	5.07 ± 3.36	0.114
ODI ^b^			
Exfuse	13.77 ± 8.93	17.58 ± 11.04	0.187
Bongener	16.34 ± 7.75	17.40 ± 6.07	0.766
Bonfuse	17.40 ± 10.76	15.60 ± 8.55	0.623
Total	15.53 ± 9.13	16.81 ± 9.14	0.497
EQ-5D ^c^			
Exfuse	0.505 ± 0.334	0.500 ± 0.307	0.961
Bongener	0.502 ± 0.267	0.400 ± 0.234	0.414
Bonfuse	0.408 ± 0.354	0.454 ± 0.344	0.710
Total	0.480 ± 0.319	0.464 ± 0.301	0.812

^a^ Visual analogue scale; ^b^ Oswestry disability index; ^c^ European Quality of life-5.

## Data Availability

Data can be obtained from the corresponding author upon reasonable request.
